# Cost-utility analysis and impact on the environment of videoconference in pressure injury. A randomized controlled trial in individuals with spinal cord injury

**DOI:** 10.1038/s41394-024-00621-w

**Published:** 2024-03-08

**Authors:** Ingebjørg Irgens, Linn Kleven, Jana Midelfart-Hoff, Rolf Jelnes, Marcalee Alexander, Johan K. Stanghelle, Tiina Rekand

**Affiliations:** 1 Sunnaas Rehabilitation Hospital, Bjørnemyrveien 11, 1450 Nesoddtangen, Norway; 2 University of Oslo, Medical Faculty, Institute of Clinical Medicine, PO Box 1171, Blindern, 0318 Oslo, Norway; 3 Norwegian Institute of Public Health, PO Box 222, Skøyen, N-0213 Oslo, Norway; 4 County Governor, Vestland, Solheimsgaten 13, 5058 Bergen, Norway; 5 VID Specialized University, Faculty of Health, 5021 Bergen, Norway; 6 Medical Center, Hospital Sønderjylland, Kresten Philipsens Vej 15, 6200 Aabenraa, Denmark; 7 Department of Physical Medicine and Rehabilitation, University of Alabama at Birmingham School of Medicine, 1670 University Blvd, Birmingham, AL 35233 USA; 8 Department of Physical Medicine and Rehabilitation, Spaulding Rehabilitation Hospital, Harvard School of Medicine, 25 Shattuck Street, Boston, MA 02115 USA; 9 Sustain Our Abilities, Jefferson Medical College, Palm Coast, FL USA; 10 Haukeland University Hospital, Department of Neurology/Spinal Cord Unit, Jonas Lies vei 71, 5053 Bergen, Norway; 11 Sahlgrenska Academy and Institute for Neuroscience and Physiology, University of Gothenburg, Box 100, S-405 30 Gothenburg, Sweden

**Keywords:** Health care economics, Quality of life, Health services

## Abstract

**Study design:**

A prospective randomized controlled trial (RCT) in persons with spinal cord injury (SCI) and ongoing pressure injury (PI).

**Objectives:**

The main aim was to perform a cost-utility analysis (CUA) alongside the RCT comparing regular care to regular care with additional videoconference consultations. Secondary aims were to assess costs and greenhouse gas emission related to transportation in the two study groups.

**Setting:**

Two spinal cord units in Norway.

**Methods:**

Participants were allocated to a regular care group (RCG) and a regular care group with additional videoconference (VCG), in a 1-year follow-up between 2016 and 2018. Costs were prospectively collected, and health-related quality of life (HRQoL) data were collected at baseline and 12 months. The outcome was quality-adjusted life years (QALYs), derived from the EQ-5D-5L questionnaire. Results are reported as incremental cost-effectiveness ratio (ICER), expressed as the cost per additional QALY gained. Transportation related costs and environmental emissions were compared by *t*-tests.

**Results:**

There were 56 participants included, 28 in each group. Of these 27 in the VCG and 26 in the RCG completed. Three participants died. The mean cost per patient was € 8819 in the VCG and € 3607 in the RCG, with 0.1 QALYs gained in the VCG. No significant differences were identified regarding HRQoL or secondary outcomes.

**Conclusion:**

The VCG costs € 5212 more for an additional 0.1 QALYs, giving an ICER of € 52,120 per QALY. No significant differences were found regarding transportation-related costs, or emission of greenhouse gases.

**Trial registration:**

www.ClinicalTrials.gov; NCT02800915, TeleSCIpi. CRISTIN.no. https://app.cristin.no/projects/show.jsf?id=545284. Sunnaas Rehabilitation hospital’s web page, available at https://www.sunnaas.no/fag-og-forskning/fagstoff/sar.

## Introduction

Spinal cord injury (SCI) is a complex, life-long condition with a high risk of developing associated conditions, such as pressure injury (PI) [1–3]. Transporting individuals with SCI and PI to the hospital may worsen the condition, hinder PI healing, and exacerbate pain and distress [2, 4].

In addition to human suffering, the costs associated with PI are considerable [1, 5–8]. Telemedicine for preventing and treating PI after SCI has shown promising results [1, 8, 9].

However, there is a lack of knowledge regarding the cost-effectiveness of telemedicine in treatment and follow-up in this particular group of patients [10].

A search in six databases identified one cost-utility study of telemedicine treatment in patients with SCI and PI. The study indicated that telephone based support had a high probability of being cost-effective given the willingness-to-pay [9]. However, the study was a short-time follow-up, carried out in low-and middle-income countries, and the results are not transferable to the healthcare service in Norway [10–12]. Two other studies concluded that considerable methodological heterogeneity made the comparison of costs and outcomes from different telemedicine studies difficult [13, 14].

In addition, environmental studies have found a positive impact of telemedicine follow-up, compared to in-person consultations, however further knowledge regarding the effect of telemedicine treatment is warranted [1, 9, 10, 15, 16].

The main aim of the current study was to perform a cost-utility analysis (CUA) to compare the costs and outcomes of regular care compared to regular care with additional videoconference consultations of PI treatment in persons with SCI. The outcome measures are costs and quality-adjusted life years (QALYs), expressed as an incremental cost-effectiveness ratio (ICER). The secondary aims were to assess the costs and greenhouse gas emission related to transportation in the two study groups. Our hypothesis was that outpatient follow-up via additional videoconference could be cost-effective and provide increased environmental sustainability.

## Material and methods

### Study design

**Fig. 1:**
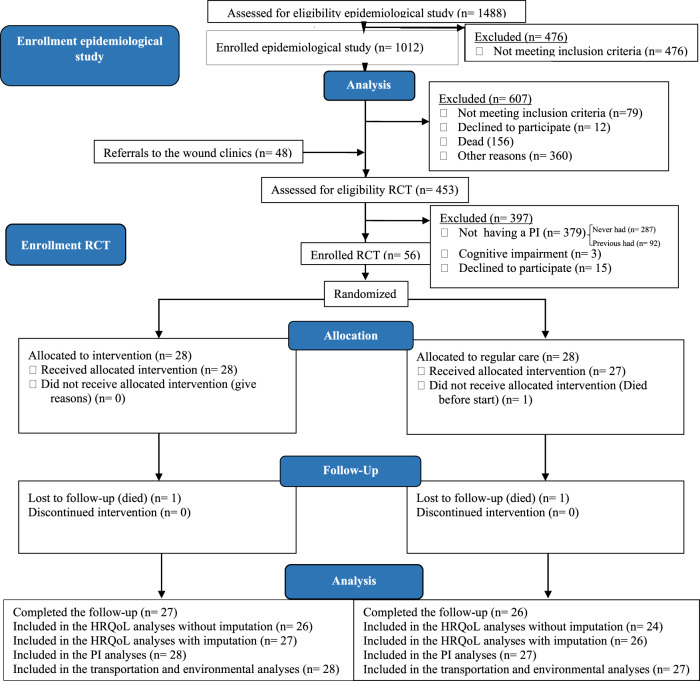
The CONSORT 2010 flow diagram of the trial. The flowchart has previously been used in a publication [8]. Copied with permission from the Journal and the author.

A CUA was conducted alongside an open-label, randomized controlled trial (RCT) at two Norwegian spinal cord units (SCUs), located at Haukeland University Hospital in Western Norway, and Sunnaas Rehabilitation Hospital in South-Eastern Norway between 2016 and 2018. Participants and their health care contacts were recruited from municipalities all over Norway. The study flowchart is shown in Fig. 1 [8].

Ethical approval was obtained from the Norwegian Regional Committee for Medical and Health Research (REK), 2014/ 684/ REK-Nord, https://www.rekportalen.no.

The CUA is reported in compliance with the Consolidated Health Economic Evaluation Reporting Standards (CHEERS) guidelines [17].

### Participants

According to Norwegian research legislation, participants are recommended to be 18 years or older and cognitively capable of giving consent to be included in a medical study [18]. Further inclusion criteria were having a SCI and ongoing PI, being a Norwegian citizen, and living in Norway at the time of participation.

Participants were recruited based on answers to a national PI-related questionnaire, and through referrals to the outpatient wound clinic at the two SCUs. Eligible participants were provided with written and oral information and signed a written consent before inclusion.

### Randomization and blinding

Participants were randomized to a regular care group (RCG), and an intervention group, followed-up via videoconference in addition to regular care (VCG). The randomization was performed by a random-number generator in the statistical software SPSS^©^, created by an external statistician. The first author participated as a physician in the follow-up, and the intervention group used a screen as mode of communication. It was difficult to blind in accordance with the group assignment; however, only the randomized number, not the identity of the participants, was known during the analysis of the results.

### Intervention

The follow-up in the RCG included outpatient on-site, telephone and ambulatory home visits, based on requests from the participants or their local healthcare contacts, according to current practices. The local health care contacts participated on-site in the telephone consultations and ambulatory home visits but did not participate in the on-site consultations at the outpatient wound clinic.

In the VCG, video consultations were offered regularly to participants in their homes, with local healthcare contacts participating on-site. The frequency of consultations was set to every second to third week, based on clinical experience and feedback from a previous feasibility study [19]. The video consultations were the intervention; however, the participants in this group were also offered regular care due to ethical issues related to for example internet difficulties.

**Fig. 2:**
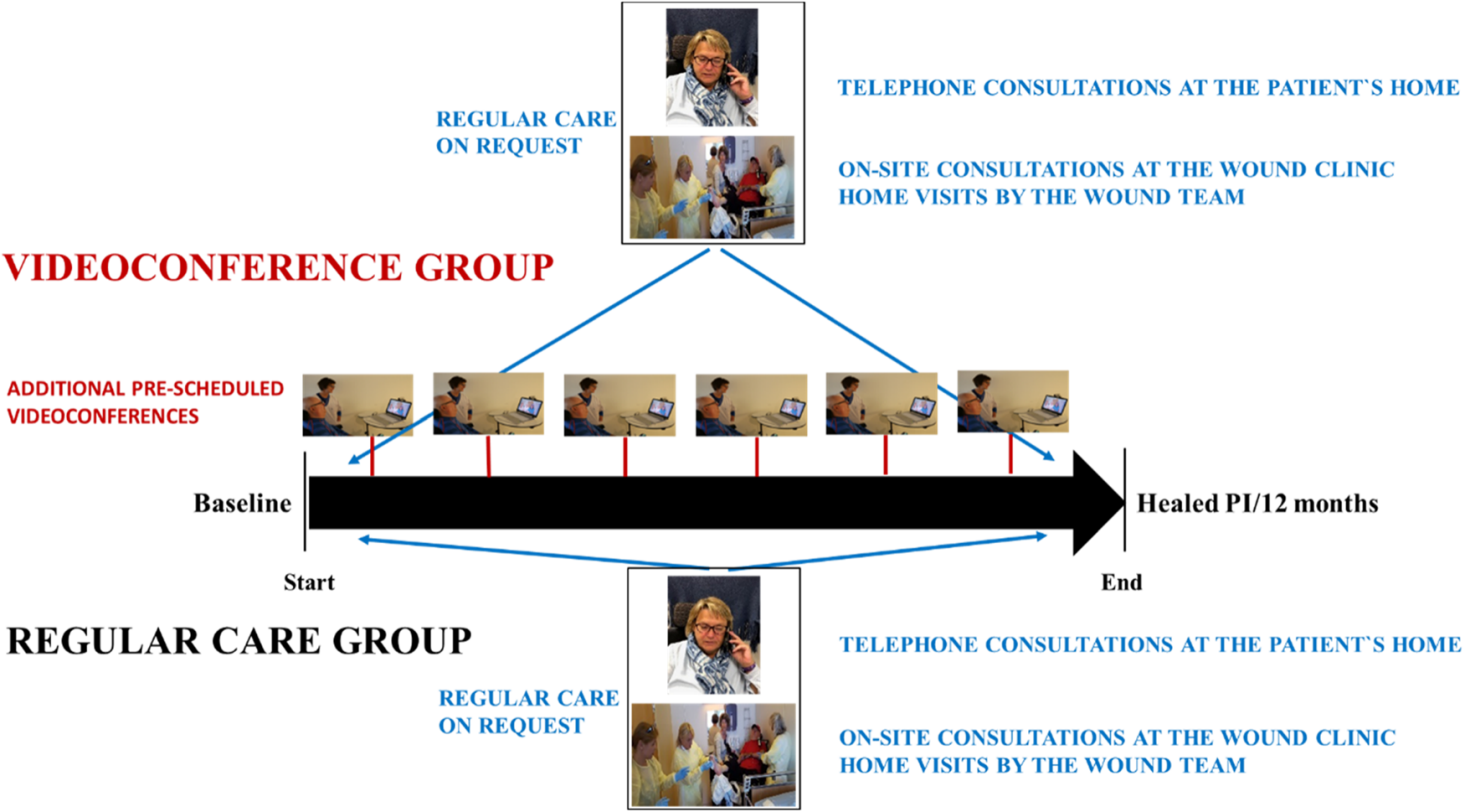
Organization of the follow-up in the two groups. The videoconference group (VCG) was offered videoconference follow-up in addition to regular care. The figure is used in a previous publication [8]. Photos and copyright by the first author, and with permission from the participants and the Journal.

Encrypted communication channels from the Norwegian Health Net (NHN) were used to protect the privacy of the participants in the VCG, in accordance with the general data protection regulation (GDPR) [20]. Figure 2 shows the intervention of the study.

Follow-up ended when the PI was healed, or after maximum 52 weeks if not healed. A previous paper describes no significant differences in the PI healing or time to healing in the two groups [8].

### Cost-utility analysis

A cost-utility analysis (CUA) of the VCG compared to the RCG was performed, adopting a healthcare perspective. The outcome measure in the analysis was quality-adjusted life years (QALYs). The QALY combines the length of life and the quality of that life into a single index, which allows for comparisons of effectiveness between the treatment groups. The results are presented as an incremental cost-effectiveness ratio (ICER), which is the difference in costs between the two groups, divided by the difference in effects (QALYs), calculated as described:

$${\rm{ICER}}=\frac{{\rm{Mean}}\,{\rm{cost}}\,({\rm{VCG}})-{\rm{mean}}\,{\rm{cost}}({\rm{RCG}})}{{\rm{Mean}}\,{\rm{effect}}\,({\rm{VCG}})-{\rm{mean}}\,{\rm{effect}}({\rm{RCG}})}=\frac{\varDelta {\rm{C}}}{\varDelta {\rm{E}}}$$

Costs are adjusted from Norwegian currency (NOK) to Euro (EUR), using the exchange rate of 2019 (€1 = NOK 9.749).

### Health-related quality of life

The HRQoL were collected at baseline and at the end of follow-up. The patient’s health state was captured using the five domains in the generic questionnaire EQ-5D-5L [21]. The responses on each domain were converted to utility weights, by using the recommended United Kingdom value set [22]. We calculated expected QALYs by multiplying the utility weights (HRQoL) with the number of life years lived in that state (1 year). The QALY results range from 0–1, where one is equal to the best imaginable health, while zero represents a health state equivalent to death. The issue of HRQoL in the two study groups has been further elaborated in a previous paper [8].

### Cost measurement

Treatment costs were collected at each consultation using a customized form. In the analysis we included costs related to the treatment (direct costs), and costs that were not directly related to the treatment (indirect costs). The consultation costs carried out by the wound team at the hospital were registered as outpatient costs, with a fixed rate.

To be able to calculate the consultation costs for the healthcare contacts in the municipalities, some assumptions had to be made in lack of exact consultation time and delay time due to technical issues, for example network failure, wound team error or patient error, during the videoconference.

In addition, we calculated costs such as toll expenses, fuel costs and ferry costs for the healthcare contacts and for the wound team during home visits. Costs of wound equipment was related to dressings used at the outpatient wound clinic. Productivity loss was not included in the analysis since most of the participants had their income from disability benefits. For participants that were in part-time employment, the consultations were scheduled as not to impact working hours. No investment in technical devices were needed to perform the videoconferences, and the encrypted software was used for free.

See Supplementary Materials 1 for cost element details.

### Secondary outcomes

The environmental analysis includes travel distance, travel time, travel costs, and emission of environmental pollutants due to travel. The Michelin Travel’s Route Planner [23] are used to calculate the roundtrip and detailed travel costs. Emission of atmospheric pollutants are calculated per kilometer (km) travel, converted to carbon oxide values, referred to as CO_2_-equivalents, and expressed in tons.

### Cost-utility analysis

The trapezoidal method (area under the curve) was used to calculate the differences in QALYs between the two treatment groups, using values from HRQoL at baseline and at the end of follow-up [24, 25]. To illustrate the statistical uncertainty surrounding the ICER, a sensitivity analysis with 1000 replications has been performed using the bootstrapping method.

Mean values together with standard deviation (SD) of HRQoL and corresponding 95% confidence interval (CI) are presented for each of the two treatment groups. Imputed values of EQ-5D-5L are used for participants who didn’t answer the end-of study HRQoL questionnaire (see Fig. 1).

The CUA values are descriptively presented, without any interferential statistics performed, in accordance with present guidelines [24, 25]. The CUA is performed using Microsoft Excel.

### Statistical analysis

Continuous variables are presented as mean with standard deviation (SD) and confidence intervals (CI), whereas categorical variables are presented as counts and percentages. Due to a low number of participants from one of the study sites, and all of them being randomized to only one of the two study groups, we did not apply multilevel analysis to account for any effect of different recruiting sites. Comparison between the two groups in the environmental analysis are performed by Mann–Whitney tests and independent samples *t*-tests.

For all the statistical analysis, *p* values <0.05 are considered significant. The analysis is performed according to the intention-to-treat principle. All statistical analysis is performed using the SPSS 26 statistical software package.

### Sample size

A sample size calculation was performed based on HRQoL. The hypothesis of this study was that HRQoL would increase in the VCG compared with the RCG. The HRQoL of individuals with SCI and PI in Norway was a relatively unexplored area, making a standard sample size calculation difficult. Thus, we decided to base the sample size calculation on Cohen’s standardized differences, avoiding the need for any assumption about, for example the variation (SD) in the data. We assumed a standardized difference of at least 0.8, which is typically considered a large effect. With 80% power and a 5% significance level, 25 participants were needed in each of the two groups, hence 28 were included to take account of some dropouts.

## Results

The study included 56 participants with acquired SCI and ongoing PI, 28 in each group. Fifty-three participants completed the study, 27 in the VCG and 26 in the RCG. One participant in the RCG died of acute illness after inclusion, but prior to finishing the baseline assessment, and the data are removed from the analysis. Baseline characteristics in the two treatments groups are shown in Table 1.

**Table 1:** Baseline characteristics of the participants.

	Videoconference group (*n* = 28)	Regular care group (*n* = 27)
Gender	*n* (%)	*n* (%)
Men	24 (86)	21 (78)
Women	4 (14)	6 (22)
AIS grade^a^	*n* (%)	*n* (%)
A	18 (64)	18 (67)
B	3 (11)	0 (0)
C	6 (21)	8 (30)
D	1 (4)	1 (4)
Age	Mean (SD)	Mean (SD)
Years	58 (14)	58 (13)
Roundtrip travel distance (Km)^b^	Mean (SD)	Mean (SD)
Participants^c^	235 (176)	387 (464)
District nurses	6.7 (7)	9.4 (12)
Roundtrip travel time (Min)	Mean (SD)	Mean (SD)
Participants	131 (102)	156 (124)
Local health care contacts	12 (11)	16 (15)
Roundtrip travel costs (Euro)	Mean (SD)	Mean (SD)
Participants	20 (19)	42 (78)
Local health care contacts	0.63 (1.8)	1.1 (1.9)
Roundtrip greenhouse gas emission (Tons)	Mean (SD)	Mean (SD)
Participants	0.04 (0.028)	0.06 (0.073)
Local health care contacts	0.0005 (0.0012)	0.001 (0.0024)
Wound team	0.04 (0.028)	0.06 (0.073)

One participant in the RCG died of an acute illness unrelated to PI, prior to start of the follow-up, and the participant’s data were excluded from the analysis.

*SCI* spinal cord injury.

^a^The American Spinal Injury Association Impairment Scale (AIS) determines the completeness of the SCI, with AIS A being the most severe and D being the less affected injury of the spinal cord. Transportation is described as one roundtrip.

^b^Km = kilometer.

^c^Travel distance, travel time and travel costs for the participants and the wound team are equal regarding one roundtrip. Costs are adjusted from NOK to EUR, using the exchange rate of 2019 (€ 1 = NOK 9.749).

### Number of consultations and health care contacts in the municipalities

The VCG had 158 more consultations compared to the RCG during the study period (464 vs. 306). The RCG had more requested home visits from the ambulatory wound team (20 vs. 6), and had more telephone guidance from the wound team, compared to the VCG (58 vs. 12). The VCG experienced a higher number of delays due to technical difficulties, such as network failure, wound team error or patient error in comparison to the RCG (94 vs. 13). The VCG had a higher number of healthcare contacts in the municipality compared to the RCG; details can be found in Table 2.

**Table 2:** Number of consultations and personnel involved in the municipalities in both groups.

	Videoconference group	Regular care group
Consultations (outpatient clinic)	*n*	*n*
Videoconference	202	–
Telephone, planned	12	58
Telephone, unplanned	226	215
Outpatient visit	18	13
Home consultations with wound team	6	20
Personnel (Municipality health service)		
One nurse	168	93
Two nurses	39	3
Nurse and occupational therapist	3	1
Health care worker	1	0
District wound nurse	28	4
Occupational therapist	11	15
Relatives	30	10
Physical therapist	2	3
Nurse and relative	18	4
Relative and assistant	0	1
Nurse and assistant	0	1
General practitioner	1	1
General practitioner and nurse	4	4
Technical problems		
Number of delays due to technical issues^a^	94	13

The costs are unevenly distributed between the multidisciplinary wound team and the health care contacts in the municipalities.

^a^Technical issues = Network failure, wound team error or patient error.

### Health-related quality of life

One participant in each of the two groups did not complete any of the HRQoL questionnaires and were removed from the HRQoL analysis. Imputed values of HRQoL were used for participants who didn’t answer the end-of-study HRQoL questionnaire (see Table 3). The mean QALYs for the participants in the VCG was 0.45 (95% CI = 0.38–0.52) vs. 0.35 (95% CI = 0.27–0.44) in the RCG, meaning no difference in HRQoL in the two groups.

**Table 3:** Total costs (direct and indirect) and HRQoL, with and without imputed values.

		**Videoconference group (*n*** = 27)			**Regular care group (*n*** = 26)	
**Direct costs**	***n***	**Mean (SD)**	**95% CI**	***n***	**Mean (SD)**	**95% CI**
Consultations (Wound team)						
Videoconference	202	6691 (4176)	(5116–8266)	0	0	0
Telephone, planned^a^	12	–	–	58	–	–
Outpatient visits	18	596 (730)	(321–872)	13	447 (712)	(178–716)
Home visits by the wound team	6	199 (372)	(58–339)	20	688 (1670)	(58–1318)
Telephone, unplanned	226	7486 (5667)	(5348–9624)	215	7395 (6658)	(4884–9907)
Delay costs, technical issues^b^	94	26 (26)	(16–35)	13	1 (2)	(−0.2–1.3)
Staff (municipality/hospital)						
One district nurse (DN)	168	241 (243)	(149–332)	93	138 (155)	(80–197)
Two DNs	39	112 (242)	(21–203)	3	9 (33)	(−3–21)
DN and occupational therapist	3	8 (31)	(−3–20)	1	3 (14)	(−3–8)
Assistant DN	1	1 (6)	(−1–3)	0	0	0
District wound nurse	28	518 (2447)	(−405–1441)	4	77 (300)	(−36–190)
District occupational therapist	11	190 (513)	(−3–384)	15	269 (596)	(45–494)
Relatives	30	292 (641)	(50–534)	10	101 (207)	(23–179)
District physiotherapist	2	35 (125)	(−12–82)	3	55 (152)	(−2–112)
DN and relative	18	512 (1784)	(−162–1185)	4	118 (349)	(−14–250)
Relative and personal assistant	0	0	0	1	25 (127)	(−23–73)
DN and personal assistant	0	0	0	1	35 (173)	(−31–100)
Physician (GP^c^ or hospital MD^d^)	1	30 (151)	(−27–86)	1	31 (153)	(−27–89)
DN and physician (GP)	4	74 (379)	(−69–217)	4	77 (386)	(−68–223)
Equipment						
Dressings^e^		321 (251)	(227–416)		178 (154)	(119–237)
Indirect costs						
Transport, roundtrips^f^						
Patients	18	23 (31)	(11–35)	25	31 (59)	(9–54)
District nurses^g^	165	103 (118)	(58–147)	5	59 (177)	(−8–125)
Wound team^h^	6	7 (16)	(1–14)	37	4 (11)	(0.3–8)
Direct costs		8687 (5088)	(6768–10,606)		3509 (2131)	(2690–4328)
Indirect costs		133 (118)	(88–178)		98 (179)	(29–167)
Total costs^i^		8820 (5184)	(6865–10,775)		3607 (2191)	(2765–4449)
**Completed EQ-5D-5L,** **without imputation**	***n***	**Mean (SD)**	**95% CI**	***n***	**Mean (SD)**	**95% CI**
At baseline	27	0.44 (0.19)	0.36–0.52	26	0.35 (0.24)	0.25–0.45
At end of follow-up	26	0.46 (0.21)	0.38–0.54	24	0.37 (0.25)	0.27–0.47
Completed EQ-5D-5L, with imputation						
At baseline	27	0.44 (0.19)	0.36–0.52	26	0.35 (0.24)	0.25–0.45
At end of follow-up	27	0.46 (0.20)	0.38–0.54	26	0.35 (0.25)	0.25–0.45

*HRQoL* health-related quality of life.

^a^Network failure, wound team error or patient error. Additional consultation time is calculated by multiplying the hourly wage for district nurses by the 10-min delay caused by the technical issue.

^b^GP = General practitioner.

^c^Hospital physician most likely the plastic or orthopedic surgeons.

^d^Costs of the different dressings used at the wound clinic.

^e^Costs of the roundtrips for the patients, the district nurses and the wound team includes toll expenses, fuel costs and ferry costs as well as travel time for each roundtrip.

^f^Travel costs and hourly wage for the district nurses multiplied with travel time in minutes.

^g^Travel costs and hourly wage for the wound team participants, multiplied with travel time in minutes.

^h^Direct and indirect costs are measured in Euros, using the exchange rate of 2019 (€1 = NOK 9.749).

^i^Total cost = direct costs plus indirect costs.

### Costs

The mean total cost per patient was €8819 (95% CI = 7727–9911) in the VCG and €3607 (95% CI = (3144–4070) in the RCG. Table 3 displays the mean total costs per individual and the HRQoL (with and without imputation) for the two groups.

The differences in mean total costs and mean difference in QALYs in the two treatment groups are summarized as an ICER, reported as the cost per QALY, and estimated to be approximately €52,120 per QALY gained (see Table 4).

**Table 4:** The incremental cost-effectiveness ratio (ICER).

	Mean costs	Incremental costs	Mean effect (QALYs)	Incremental effect (QALYs)	ICER (Cost/Effect)
Regular care (RCG)	€ 3607		0.35	0.1	€ 52,120
Videoconference (VCG)	€ 8819	€ 5212	0.45		

The ICER is calculated as the difference in costs per patient divided by the difference in effects (QALYs) per patient, based on the completed EQ-5D-5L with imputation.

**Fig. 3:**
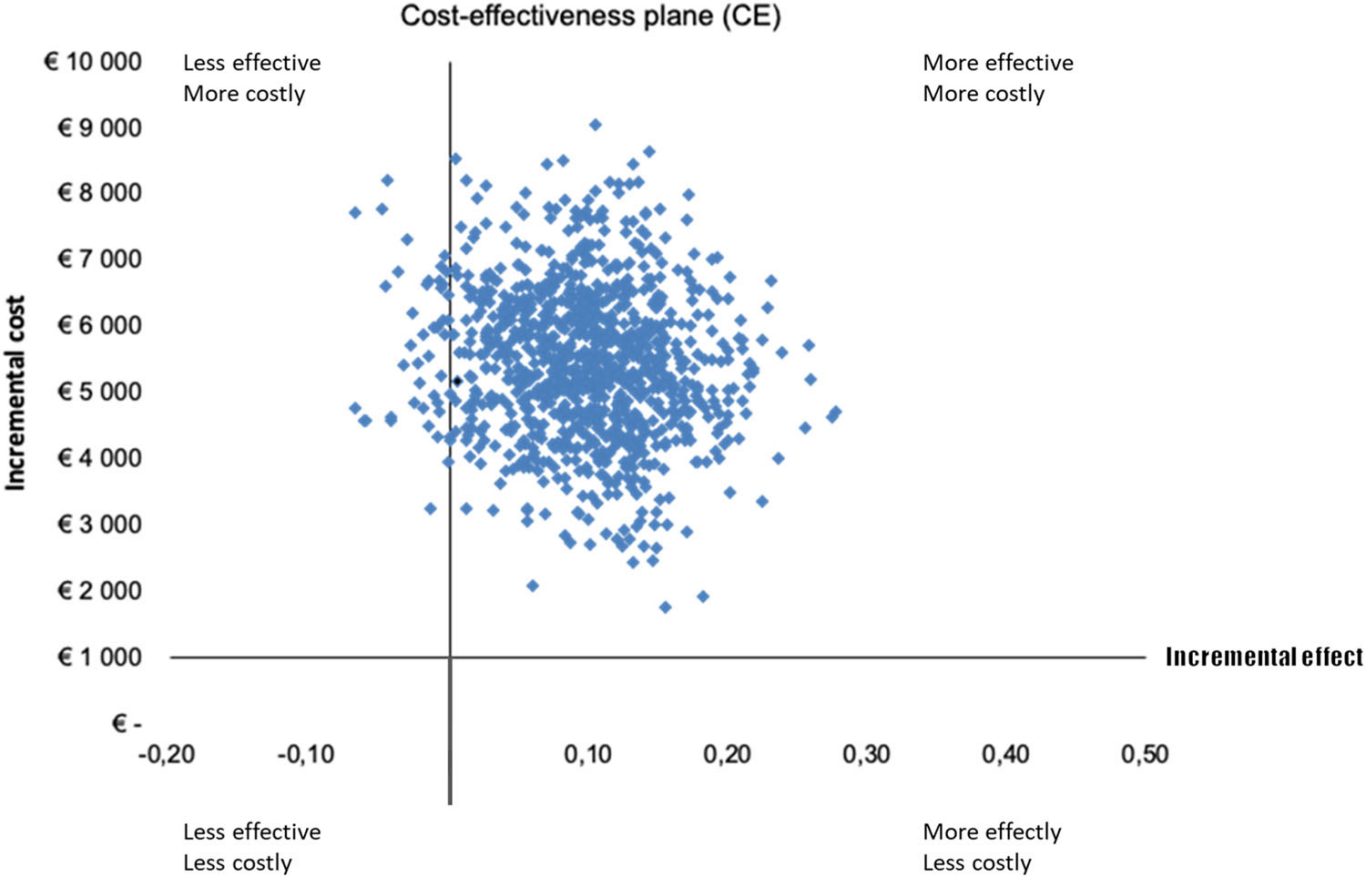
The cost-effectiveness (CE) plane displays incremental effects (QALYs with imputation) on the *x*-axis and incremental costs (mean direct and indirect costs) on the *y*-axis. The CE-plane visually represents the differences in costs and effects (QALYs) between the two treatment groups after 1000 bootstrap replications.

Uncertainty in the incremental cost and effects are illustrated by a scatterplot on a cost-effectiveness plane (CE) (see Fig. 3).

#### The environmental analysis

No significant differences were found for travel distance (mean difference −29.8, with 95% CI = −152.3–92.7, *p* value = 0.627), travel time (mean difference 45.2, with 95% CI = −36.5–126.9, *p* value = 0.272), travel costs (mean difference −2.840, with 95% CI = −11.0–5.3, *p* value = 0.490) or emission of CO_2_ equivalents (mean difference −0.010, with 95% CI = −003–0.0080, *p* value = 0.266). Supplementary Materials 2 gives details regarding the secondary outcomes.

A scenario analysis was modeled, comparing videoconference treatment only, with on-site consultations only, based on the actual number of consultations in the two groups. In this analysis, all results were significant in favor of videoconference consultations The modeled comparison between the two groups is shown in Supplementary Materials 3.

## Discussion

The current study explores the potential health outcomes and associated costs of introducing videoconference consultations in addition to regular care (VCG) when compared to regular care alone (RCG).

During the 1-year follow-up, virtually no difference in health-related quality of life was observed between the two groups. In a clinical setting, the patients will not improve, nor decrease their quality of life by being followed-up via videoconference consultations. However, higher costs were estimated in the VCG. In Norway, there is no exact cost-effectiveness threshold (willingness-to-pay), leading to uncertainty regarding the maximum amount a stakeholder is willing to pay for a unit of health outcome (one QALY). However, cost estimates range from €28,208 per QALY at the lower limit to €84,624 per QALY in the higher severity class. The willingness-to-pay threshold varies based on the severity of the disease or condition; the more severe the condition, the higher the acceptable cost per QALY [26].

The number of participating health care professionals from the municipalities was higher in the VCG compared to the RCG, however the personnel costs constituted less of the direct costs in the VCG compared to the RCG. Thus, the main difference in costs between the two groups was the number of pre-planned video consultations in the VCG. The frequency of consultations in the VCG was set to once every second to third week, based on experience and feedback from a feasibility study conducted as a pilot before the present study [19]. This possibly increased the number of consultations in the VCG above what was required, based on no significant differences in the PI healing or time to healing in the two groups, as presented in a previous paper [8].

The increased number of attending local health personnel in the VCG is probably partly explained with a need for more personnel at the video consultations to learn to use the digital technology. However, more attending staff also implies more widespread knowledge about SCI and PIs and a chance for better co-operation across different healthcare levels and has a positive effect not only for the actual patient, but also for the community in general [1–3, 5, 9–12].

The number of technical issues in the VCG was increased compared to the RCG. Most of these issues were related to software change at the network provider, a change that entailed a period of decreased internet connection, and thus a need to compensatory increase the number of unplanned telephone consultations in the VCG. These issues were temporary and will probably not affect future studies. However, the delay costs due to technical issues in the current study increased the costs in the VCG vs. the RCG.

The number of telephone consultations on request in the RCG were almost three times the number in the VCG. In the current study, video consultations provided a visual advantage compared to telephone consultations, enabling remote visual examination and feedback of the PI, as well as maintaining visual contact with participants during the consultation. Our experience suggest that this provided health personnel with a more effective tool for making the right treatment decisions [1–3].

Previous telemedicine research has yielded inconsistent findings in costs, largely due to different care models, thus it is difficult to directly compare the outcomes in the present study [2, 5, 6, 9, 10, 12–16]. Further, in the current study, the team-based approach to treatment of PIs is influenced by Norway being a high-income country, making direct comparison with studies performed in low- and middle-income countries challenging [9–14].

The occurrence of severe PIs often leads to long periods of hospitalization and frequent outpatient follow-ups to the hospital to monitor the treatment [2, 3, 5, 6]. The current study did not find any significant differences in transportation-related costs in the two groups, however, the modeled analysis showed significant better results in favor of the VCG in terms of travel distance, travel time and travel costs. In Norway, transportation is generally accessible, but climate change has interfered with the seasons, and the weather has become worse. In some cases, the weather conditions may result in cancellation of on-site consultations. Further, pandemics, such as Covid-19, may reduce on-site activity in the outpatient clinics, making it necessary for healthcare professionals to think creatively about follow-up possibilities.

Expenses associated with transportation to the hospital are high in terms of time and money, and as long as fossil fuels are used, the expenses are also high in terms of the carbon footprint [27]. The modeled analysis in our study found significant better results regarding emission of greenhouse gases. Reduced transport to/from appointments at the hospital would contribute to less emission of atmospheric pollutants. Positive environmental effects of remote follow-up, as compared to in-person consultations at the outpatient clinic has been documented [27, 28].

According to the United Nation’s sustainability development goal regarding climate action, all parts of the society should work to reduce their carbon footprints [29], and as such, videoconference has the potential to improve the climate sustainability.

### Limitations

Three participants were excluded from the HRQoL analyses, due to lack of information at baseline and end-of follow-up, thus a modified intention-to-treat analysis was performed. Excluding patients from an intention-to-treat randomized trial will increase the risk of selection bias, however, the exclusion did not change the distribution of participants in the two groups [30].

It was not possible to compare videoconference consultations only to on-site consultations only, due to ethical and clinical considerations, as well as the choices made by participants regarding their follow-up. Thus a hybrid, personalized solution of follow-up, may have the opportunity to offer more sustainable healthcare options.

The low number of participants in the study makes it difficult to generalize the results to the total population, even though the number was not limited by strict inclusion and exclusion criteria.

The study has low power regarding the other outcomes investigated, and the assumptions regarding potential differences in HRQoL in the two groups were too optimistic. A non-inferiority design would have been better to establish knowledge regarding if the additional videoconference follow-up was no worse than the traditional offer. However, this was not possible to perform due to the large number of participants needed.

The analysis lacks an evaluation of costs associated with benefits realization, such as patient empowerment, collaboration, and knowledge transfer, which is, however warranted. Conducting such analysis would provide valuable insights for making informed decisions on prioritizing telemedicine in the follow-up of individuals with complex long-term needs.

## Conclusion

The cost-utility analysis demonstrated that VCG was more costly compared to RCG, with € 52,120 per QALYs gained. The willingness-to-pay thresholds are not precisely defined in Norway, making it uncertain if VCG falls within an acceptable range. No significant differences were found in the two groups regarding transportation-related costs, or emission of greenhouse gases.

### Supplementary information

Supplementary materials 1

Supplementary materials 2

Supplementary materials 3

## Data Availability

The dataset is stored in a locked and fireproof research cabinet at the research department, Sunnaas Rehabilitation Hospital, Norway, and can be made available on request according to the Norwegian Data and Telecommunications Authority’s requirements for safe information flow.
