# Supplementary material for: Cost-utility analysis and impact on the environment of videoconference in pressure injury. A randomized controlled trial in individuals with spinal cord injury
Source: Spinal Cord Ser Cases. 2024 Mar 8;10:10. doi: 10.1038/s41394-024-00621-w (PMC10923859; doi:10.1038/s41394-024-00621-w)
Supplement: Supplementary file 1 — Supplementary materials 1 [file 41394_2024_621_MOESM1_ESM.docx]

**Supplementary materials 1. Identification, valuation and quantification of the different cost elements used in the cost-utility analysis**

| **Cost elements** | **Unit** | **Valuation** | **Cost (EUR)** | **Source** |  |
| --- | --- | --- | --- | --- | --- |
|  |  |  |  |  | |
| ***Consultation (Wound team)*** |  |  |  |  | |
| Videoconference | Per patient | Cost | € 894 | Sunnaas Rehabilitation Hospital^1^ | |
| Outpatient visit | Per patient | Cost | € 894 | Sunnaas Rehabilitation Hospital | |
| Home visits | Per patient | Cost | € 894 | Sunnaas Rehabilitation Hospital | |
| Telephone | Per patient | Cost | € 894 | Sunnaas Rehabilitation Hospital | |
| ***Personnel (the municipal health service)*** |  |  |  |  | |
| District nurse | Hours | Wages | € 46 | Statistics Norway^2^ | |
| Occupational therapist | Hours | Wages | € 43 | Statistics Norway | |
| District wound nurse | Hours | Wages | € 46 | Statistics Norway | |
| Assistant nurse (Health care worker) | Hours | Wages | € 39 | Statistics Norway | |
| Personal assistant | Hours | Wages | € 37 | Statistics Norway | |
| Relatives | Hours | Wages | € 24 | The Norwegian Medicines Agency^3^ | |
| Physical therapist | Hours | Wages | € 44 | Statistics Norway | |
| General practitioner | Hours | Wages | € 74 | Statistics Norway | |
| ***Wound equipment*** |  |  |  |  | |
| Dressing |  |  |  |  | |
| Category 1 | Bandages | Costs | € 10 | Sunnaas Rehabilitation Hospital | |
| Category 2 | Bandages | Costs | € 21 | Sunnaas Rehabilitation Hospital | |
| Category 3 | Bandages | Costs | € 31 | Sunnaas Rehabilitation Hospital | |
| 1 Finance department at Sunnaas Hospital (SunHF) | |  |  |  | |
| 2 SSB: Statistics Norway; earnings (statistikkbanken) | |  |  |  | |
| 3 The Norwegian Medicines Agency, unit costs database | |  |  |  | |
