# Supplementary material for: Cost-utility analysis and impact on the environment of videoconference in pressure injury. A randomized controlled trial in individuals with spinal cord injury
Source: Spinal Cord Ser Cases. 2024 Mar 8;10:10. doi: 10.1038/s41394-024-00621-w (PMC10923859; doi:10.1038/s41394-024-00621-w)
Supplement: Supplementary file 2 — Supplementary materials 2 [file 41394_2024_621_MOESM2_ESM.docx]

**Supplementary materials 2. Comparison of the accurate transportation costs in the two groups**

|  | **Videoconference** | | **Regular care** | | |  | **Comparison** |  |
| --- | --- | --- | --- | --- | --- | --- | --- | --- |
|  |  |  |  |  | |  |  |  |
|  | **Mean (SD)** | **95 % CI** | **Mean (SD)** | **95 % CI** | **Mean diff.** | | **95 % CI** | **p-value** |
|  |  |  |  |  | |  |  |  |
| Travel distance (Km) | 159.2 (157.8) | 98.0 to 280.4 | 189.0 (280.4) | 78.1 to 300.0 | | -29.8 | -152.3 to 92.7 | 0.627 |
| Travel time (Min) | 175.3 (164.3) | 111.6 to 239.0 | 130.1 (135.8) | 76.4 to 183.9 | | 45.2 | -36.5 to 126.9 | 0.272 |
| Travel costs (€) | 10.9 (11.8) | 6.3 to 15.5 | 13.7 (17.9) | 6.63 to 20.8 | | -2.84 | -11.0 to 5.3 | 0.490 |
| Atmospheric pollutant emission (Tons) | 0.019 (0.019) | 0.013 to 0.027 | 0.029 (0.044) | 0.012 to 0.047 | | -0.010 | -0.03 to 0.0080 | 0.266 |

^A comparison of videoconference treatment in addition to regular care with regular care, based on the actual number and type of consultations in the two groups. The mean difference is the mean in the videoconference group minus the mean in the regular care group. SD= Standard deviation, CI= confidence interval, Km= kilometre, Min= minutes, €= Euro.^
