# Supplementary material for: Cost-utility analysis and impact on the environment of videoconference in pressure injury. A randomized controlled trial in individuals with spinal cord injury
Source: Spinal Cord Ser Cases. 2024 Mar 8;10:10. doi: 10.1038/s41394-024-00621-w (PMC10923859; doi:10.1038/s41394-024-00621-w)
Supplement: Supplementary file 3 — Supplementary materials 3 [file 41394_2024_621_MOESM3_ESM.docx]

**Supplementary materials 3. A modelled scenario comparing the transportation costs in videoconference consultations only and on-site consultations only**

|  | **Videoconference** | | **Regular care** | | |  | **Comparison** |  |
| --- | --- | --- | --- | --- | --- | --- | --- | --- |
|  |  |  |  |  | |  |  |  |
|  | **Mean (SD)** | **95 % CI** | **Mean (SD)** | **95 % CI** | **Mean diff.** | | **95 % CI** | **p-value** |
|  |  |  |  |  | |  |  |  |
| Travel distance (Km) | 64.8 (99) | 26.5 to 103 | 1440.7 (2099) | 610.5 to 2270.8 | | -1376 | ( -2172) to (-580) | **0.001** |
| Travel time (Min) | 125.1 (99) | 61.3 to 189 | 532.8 (565) | 309.2 to 756.3 | | -407.7 | (-631) to (-184) | **0.001** |
| Travel costs (€) | 3.58 (8.34) | 0.35 to 6.81 | 170.7 (345) | 34.3 to 307.2 | | -167.2 | (-298 to -36.5) | **0.013** |
| Atmospheric pollutant emission (Tons) | 0.004 (0.0098) | 0.001 to 0.008 | 0.225 (0.329) | 0.095 to 0.355 | | -0.221 | (-0.346) to (-0.097) | **0.001** |

^A modelled scenario analysis comparing videoconference treatment only, with regular care in the form of on-site consultations only, based on the actual number of consultations in the two groups. SD= Standard deviation, CI= confidence interval, Km= kilometre, Min= minutes, €= Euro. The mean difference is the mean in the videoconference group minus the mean in the regular care group.^
